# Identification and analysis of the *β-catenin1* gene in half-smooth tongue sole (*Cynoglossus semilaevis*)

**DOI:** 10.1371/journal.pone.0176122

**Published:** 2017-05-10

**Authors:** Ying Zhu, Qiaomu Hu, Wenteng Xu, Hailong Li, Hua Guo, Liang Meng, Min Wei, Sheng Lu, Changwei Shao, Na Wang, Guanpin Yang, Songlin Chen

**Affiliations:** 1 College of Marine Life Science, Ocean University of China, Qingdao, China; 2 Laboratory for Marine Fisheries Science and Food Production Processes, Qingdao National Laboratory for Marine Science and Technology, Qingdao, China; 3 Yangtze River Fisheries Research Institute, Chinese Academy of Fishery Sciences, Wuhan, Hubei, China; 4 Yellow Sea Fisheries Research Institute, Chinese Academy of Fishery Sciences (CAFS), Key Lab for Sustainable Development of Marine Fisheries, Ministry of Agriculture, Qingdao, China; University of Kentucky, UNITED STATES

## Abstract

β-catenin is a key signalling molecule in the canonical Wnt pathway, which plays a role in cell adhesion, embryogenesis and sex determination. However, little is known about its function in teleosts. We cloned and characterized the full-length *β-catenin1* gene from half-smooth tongue sole (*Cynoglossus semilaevis*), which was designated *CS-β-catenin1*. The *CS-β-catenin1* cDNA consists of 2,346 nucleotides and encodes a protein with 782 amino acids. Although *CS-β-catenin1* was transcribed in the gonads of both sexes, the level was significantly higher in ovaries compared to testes. Furthermore, the mRNA level of *CS-β-catenin1* was significantly upregulated at 160 days and constantly increased until 2 years of age. *In situ* hybridization revealed that *CS-β-catenin1* mRNA was mainly localized in oocyte cells, especially in stage I, II and III oocytes. When *CS-β-catenin1* expression was inhibited by injection of quercetin in the ovaries, levels of *CS-Figla* and *CS-foxl2* mRNA were significantly down-regulated, and *CS-dmrt1* was up-regulated, which suggested that *CS-β-catenin1* is a potential upstream gene of *CS-Figla* and is involved in the development of the ovaries, i.e., folliculogenesis.

## Introduction

As the key molecule of cellular junctions, as well as the pivotal element of the canonical Wnt signalling pathway [1,2], β-catenin plays critical roles in cell growth and development, disease pathogenesis and cell adhesion [3,4]. Several studies have found that β-catenin has a significant link to mammalian sex determination and differentiation. In humans and mice, *β-catenin* is transcribed in the testis and ovaries [5–7], which suggests that it has a dual role in the gonad development of both sexes. Furthermore, the disruption of *β-catenin* in mouse ovaries causes severe deficiencies in gonad development, affecting the occurrence of masculinization, formation of testis-specific coelomic vessels and even the loss of female germ cells [7]. In contrast, testicular development appears to be normal regardless of mutations in *β-catenin* [7], which implies that β-catenin is necessary for ovary differentiation but seems to be dispensable for testicular development. Furthermore, *β-catenin* was found to be able to regulate the expression of many sex-related genes in mammalian ovaries. For example, β-catenin has been shown to regulate the transcription of *Cyp19a1* via interactions with NR5A1 in rat ovarian granulosa cells [8] and repress Sertoli cell-specific *Sox9* gene expression in mouse ovaries [9]. In addition, it was recently documented that activation of the canonical Wnt/β-catenin pathway in XY gonads of mice effectively blocked the male pathway and led to male to female sex reversal [5, 6]. In subsequent studies, the RSPO1/β-catenin signalling pathway was found to promote the meiosis of germ cells in the ovaries because the mutation resulted in impaired meiosis and down-regulated the expression of the early meiosis marker gene *Stra8* [10]. Consequently, the studies described above suggest that β-catenin plays an essential role in ovary determination and differentiation in mammals. In teleosts, functional homologues of *β-catenin* have only been characterized in a few species, such as *Danio rerio* and *Nile tilapia*. Interestingly, in most teleosts, two subtypes of *β-catenin* were found, which were titled *β-catenin1* and *β-catenin2* [11, 12]. The two *β-catenin* subtypes displayed similar expression patterns in the gonads and functional similarity during development. For example, *Nile tilapia β-catenin1* and *β-catenin2* are both mainly expressed in the oogonia and oocytes of the ovaries, and knocking down these genes in the ovaries causes similar gonad phenotypes, including the inhibition of ovarian differentiation and masculinization [12].

The half-smooth tongue sole (*Cynoglossus semilaevis*) is a marine fish species with economic importance, It is generally distributed throughout the China Sea and is famous for its pleasant taste. Since its genome was deciphered, the half-smooth tongue sole has become an excellent model to study sex determination and differentiation in fish. Due to obvious growth dimorphism (females grow 2–4 times faster than males) [13], cultivating an all-female stock would be greatly beneficial for aquaculture. To achieve this goal, understanding the mechanisms of sex determination and sex differentiation is necessary [14, 15]. The half-smooth tongue sole has a ZW/ZZ sex chromosome system including male (ZZ) and female (ZW) sexes. Approximately 14% of cultivated populations comprise of neomales, which are sex-reversed from genetic females (ZW) to the phenotypic males under specific environmental conditions, such as high temperature [16, 17]. Neomales show similar growth patterns as males, which makes them disadvantageous to productivity. Therefore, elucidating the sex-reversal mechanism of neomales, and especially the identification of key genes in the reversal process, is both important for theoretical study and practical applications [18, 19]. Despite the isolation of several sex-related genes, such as *dax1*, *foxl2*, *sox9a*, *amh*, *wnt4a*, *follistatin* and *cyp19a1a* [20], the mechanisms of sex determination and differentiation in this species are still unclear. In the present study, we cloned and characterized a homologue of *β-catenin1* in half-smooth tongue sole, then detected its pattern of tissue expression and temporal expression in gonads. We also investigated the potential pathways that are regulated by *CS-β-catenin1* by treating ovaries *in vivo* with quercetin. Additionally, we evaluated the correlation between promoter methylation and *CS-β-catenin1* expression. Our aims were to investigate the detailed functions of *β-catenin1* in *C*. *semilaevis* and to further reveal its role in gonad development.

## Materials and methods

### Ethical statement

All experimental animal protocols were approved by Yellow Sea Fisheries Research Institute’s animal care and use committee. All tissues were removed under MS222 anesthesia, and all efforts were made to minimize fish suffering.

### Experimental animals and sample collection

The half-smooth tongue sole used in this study were obtained from the Haiyang High-Tech Experiment Base (Haiyang, Shandong Province, China), and genetic and phenotypic sexuality was determined by previous method[13, 20]. Eight samples of each category; including the male, female and neomale, were randomly selected for sampling (three individuals of each gender).Thirteen tissues (heart, gill, liver, skin, kidney, blood, brain, spleen, muscle, pituitary, intestine, ovary and testis) were collected from 1-year post-hatching (yph) fish and immediately transferred into liquid nitrogen and stored at −80°C. For gonad samples at different developmental stages, three individuals at 7, 25, 48, 160 days post-hatching (dph), 8 and 10 months post-hatching (mph), 1 and 2 years post-hatching (yph) were sampled within one family. Simultaneously, parts of the gonads from 1 yph fish were fixed in 4% paraformaldehyde (pH 7.5) at 4°C for 20 h, then stored in 75% ethanol. In addition, parts of the caudal fins were collected and fixed in 100% ethanol for DNA extraction to determine the genetic sex of the fish.

### Isolation of full-length *CS-β-catenin1* cDNA

Total RNA was extracted from the tissues using TRIzol Reagent (Invitrogen, Carlsbad, CA, USA) according to the manufacturer’s instructions. RACE-ready first-strand cDNA was synthesized from the total RNA using a SMART™ RACE cDNA amplification kit (Invitrogen). To obtain the full-length cDNA sequence, specific primers for the outer and nest amplifications were designed based on the partial cDNA sequence of *CS-β-catenin1* from the genome sequence. The 5’-RACE and 3’-RACE reactions were performed using a SMART-RACE cDNA Amplification Kit (Clontech Inc., Mountain View, CA, USA). The specific procedures refer to Hu’s description [21].

### Quantitative real-time PCR (qRT-PCR)

Total RNA from various tissues and gonads at different developmental stages was extracted using TRIzol Reagent as described previously. The cDNA was synthesized using a QuantScript RT kit (TaKaRa, Dalian, China) following the manufacturer’s protocol. Quantification was performed on a 7500 detection system (Applied Biosystems, Foster City, CA, USA) with SYBR Green Master Mix (TaKaRa BioInc). The qRT-PCR amplification was performed as previously described [22]. *β-Actin* was confirmed previously as a reliable gene in various tissue samples of *C*. semilaevis and was used as an internal control [23]. Each sample was analysed in triplicate, and three samples were handled. The relative mRNA expression of target genes was calculated with the 2^−△△Ct^ method as described previously [24]. Data was analysed by one-way analysis of variance (ANOVA) followed by Duncan’s multiple comparisons test using SPSS 18.0 (IBM, New York, NY, USA). Using a t-test, significance was set at *P*<0.05.

### *In situ* hybridization (ISH)

To synthesize digoxigenin (DIG)-labelled RNA sense and antisense probes, we designed the primer pairs *CS-β-catenin1*-ISH-F and *CS-β-catenin1*-ISH-R (S2 Table). A 344 bp cDNA fragment from the open reading frame (ORF) region of *CS-β-catenin1* was amplified using the primer pairs above then cloned into pBluescript II SK (+) and sequenced. The recombinant plasmid was linearized by *EcoR*I and *Hind*III and was used as the template for *in vitro* synthesis of the probes. The probes were synthesized with a DIG RNA labelling kit (Roche, Mannheim, Germany) following the manufacturer’s instructions. Gonad samples were processed in a series of ethanol gradients and embedded in paraffin wax, and 5μm sections were cut. Meanwhile, three samples were analysed with ISH which was performed using the labelled probes as previously described [25].

### Expression of *CS-β-catenin1*, *CS-Figla*, *CS-Wnt4a*, *CS-Foxl2* and *CS-dmrt1* in ovaries after treatment with quercetin

Since quercetin acts as a potent inhibitor of the transcriptional activity of β-catenin [26, 27], it was selected to repress *β-catenin* expression. For the experiments, quercetin was diluted in 5% DMSO and injected into the gonads of 1 yph fish with female cavities at a dosage of 25 mg/kg/day. The dose was selected based on a previous study [28]. The injections were given once every 24 hours, and the fish were injected twice. The control groups had three individuals treated with an equal volume of 5% DMSO. The transcription levels of *CS-β-catenin1* were detected using qRT-PCR as described above. For analysis, the mRNA of these genes was examined in 1 yph ovaries.

### Analysis of *CS-β-catenin1* methylation

Most gonadal DNA methylation occurs in half-smooth tongue sole at the first exon or promoter region of the gene. To determine the epigenetic regulation of *β-catenin1*, the data for methylation levels among females, males, and neomales were compared using information about the *C*. *semilaevis* methylome [16].

## Results

### Isolation and characterization of *CS-β-catenin1* cDNA

**Fig 1:**
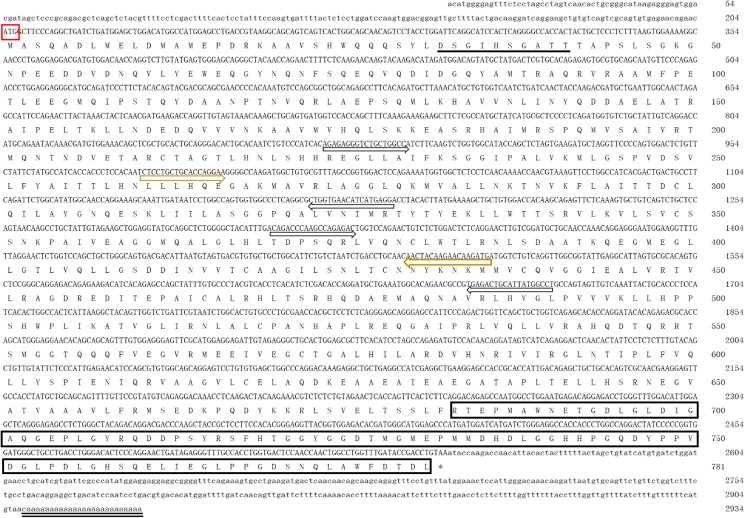
Full-length cDNA sequence and the deduced amino acids of *CS-β-catenin1*. Lowercase text indicates the 5’ and 3’UTR sequences of *CS-β-catenin1*; uppercase text indicates the coding sequence. The start codon (ATG) is in the red box. The putative ARM repeat regions and VATPaseH superfamily domain are marked with yellow arrows and black arrows, respectively. The N-terminal putative GSK-β consensus phosphorylation site is underlined, and the C-terminal transactivator region is in the box with light lines. The stop codon (TAA) is indicated by an asterisk, and the poly (A) tail is double-underlined.

A 2934 bp *CS-β-catenin1* cDNA was obtained and identified after RT-PCR and RACE (GenBank accession number KX898023). The complete cDNA coding sequence contained a 204 bp 5’UTR, a 384 bp 3’UTR, and a 2346 bp ORF that encoded a 781-amino-acid protein. Structural analysis revealed that the *CS-β-catenin1* protein could be divided into an N-terminal region, a central region and a C-terminal region. The central domain contained 12 armadillo (ARM) repeats and a cellulose synthase-interaction domain (Fig 1 and S1 Fig), and the ARM has been implicated in mediating protein-protein interactions.

### Alignment and phylogenetic analysis

**Fig 2:**
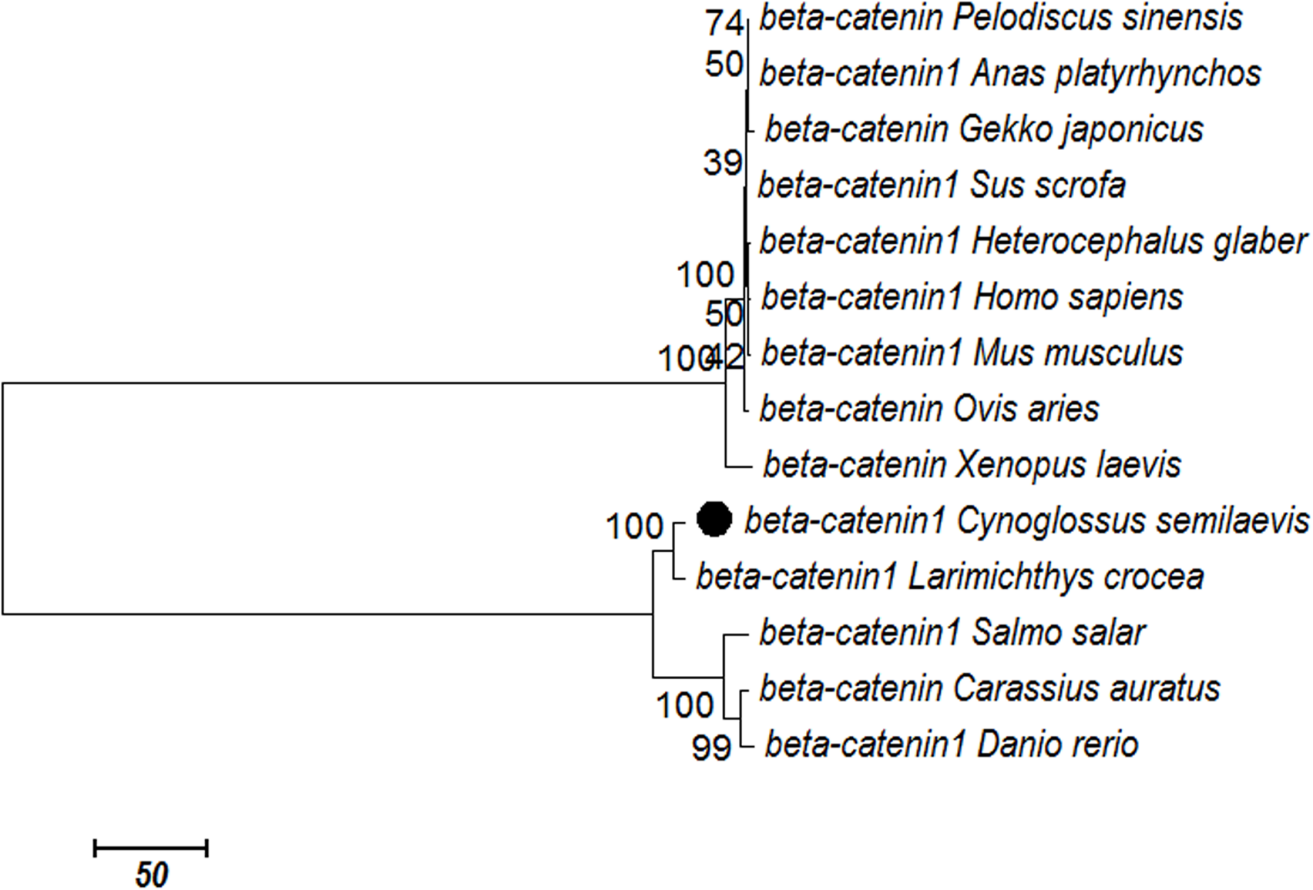
Phylogenetic analysis of the β-catenin protein among different species. The phylogenetic tree was constructed with the neighbour-joining algorithm in MEGA7.0. The bootstrap values were based on 10,000 resampling replicates. The relative genetic distances are indicated by the scale bar and branch lengths.

Through the BLASTx program, the C*S-β-catenin1* protein showed a high degree of identity to other fish, including *Larimichthys crocea* (99%), *Carassius auratus (*96%*)*, and *Pelodiscus sinensis (*96%*)*. A phylogenetic tree was constructed using *β-catenin1* from half-smooth tongue sole and 13 other species (Fig 2 and S1 Table). The sequences were clustered in two main groups, including *β-catenin* of the teleosts clustered in one group, and those of mammals clustered in another. Among the clusters, *CS-β-catenin1* was more closely related to *β-catenin* in four types of fish.

### Tissue expression of *CS-β-catenin1*

**Fig 3:**
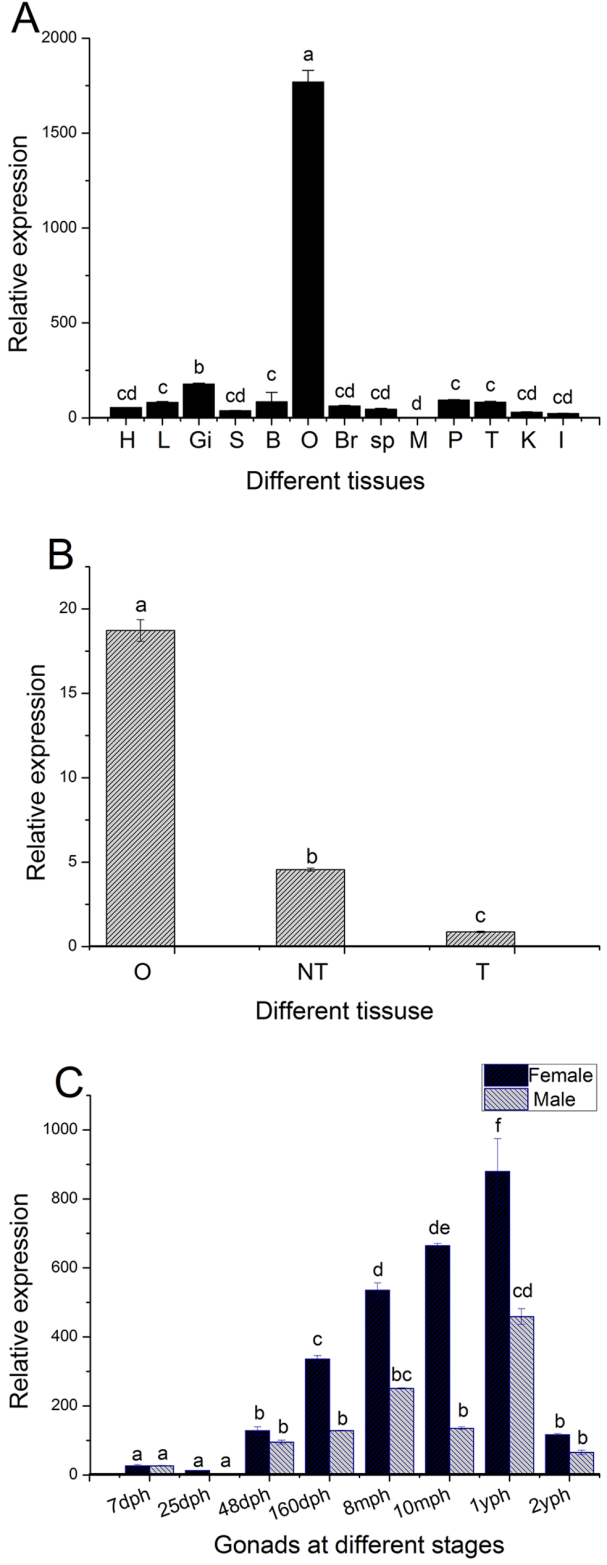
Expression levels of *β-catenin1* mRNA in *C*. *semilaevis* evaluated with qRT-PCR. (A) Expression of *CS-β-catenin1* in various tissues. H: heart, L: liver, K: kidney, I: intestine, SP: spleen, S: skin, M: muscle, B: blood, Br: brain, Gi: gill, P: pituitary, O: ovary, T: testis. (B) Expression of *CS-β-catenin1* in different genotypes. O: ovary, T: testis of males, NT: testis of neomales. (C) Expression of *CS-β-catenin1* in gonads at different developmental stages. *β-actin* was used as an internal control to normalize the expression. Three samples were performed. Data are shown as means ± SEM. The variance of this expression was represented as a ratio (the amount of *CS-β-catenin1*mRNA normalized to the corresponding reference genes values). Values with superscripts indicate statistical significance (P<0.05)

*CS-β-catenin1* expression was analysed in a wide range of tissues, e.g., the, heart, gill, brain, and liver. It is worth emphasizing that the mRNA expression of *CS-β-catenin1* detected in the ovaries was the highest, whereas the lowest levels were found in muscles. The remaining tissues that were examined had very low expression (Fig 3A). Furthermore, we detected expression in the ovaries, male testis, and neomale testis of 1 yph fish. The highest levels were observed in the ovaries, followed by expression in the testis, and lowest expression was found in the neomale testis (Fig 3B).

### *CS-β-catenin1* expression in developmental stages of male and female gonads

The expression profile of *CS-β-catenin1* was detected by qRT-PCR, and the *CS-β-catenin1* expression in the development of gonads was diverse. In the development of the female gonads, extremely low levels were observed 7 dph. Starting at 48 dph, the expression increased evidently and continued to rise until 1 yph, where peak levels were attained, and then there was a significant decline at 2 yph (Fig 3C). In the developmental stages of the testis, we detected the lowest amount of expression at 7 dph. From 48 dph to 2 yph, high expression was only observed at 8 mph and 1 yph (Fig 3C). In comparison, expression in females was higher compared to males at all tested time points except at 7 dph, when the levels were nearly equal.

### Cyto-location of *CS-β-catenin1*

**Fig 4:**
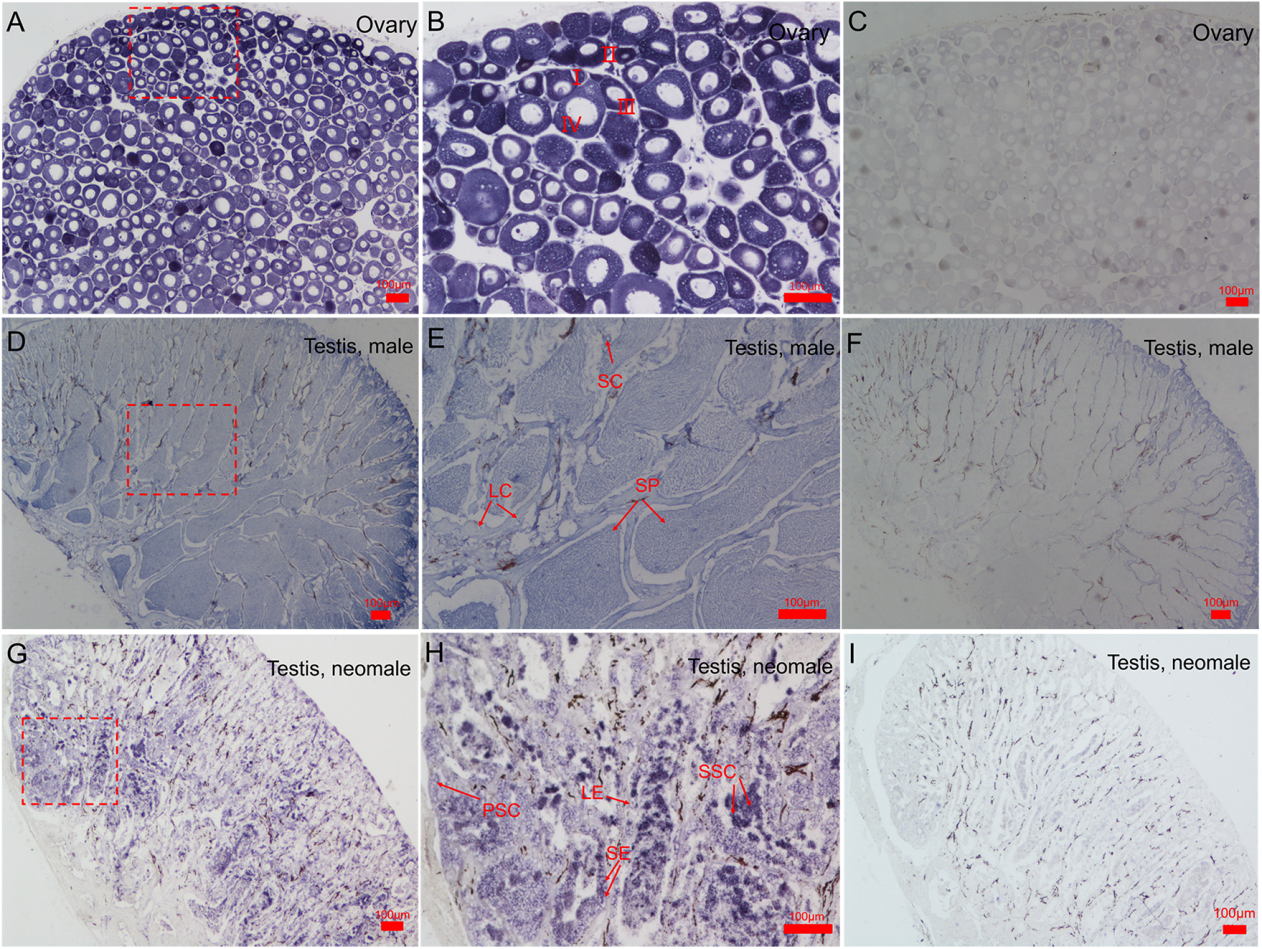
Cyto-locations of *CS-β-catenin1* mRNA in gonads of *C*. *semilaevis*. (A). Low magnification showing the adult ovaries. (B). High magnification of the framed area in (A). (C). Control of *β-catenin1* localization in female ovaries. (D). Low magnification showing the male adult testis of males. (E). High magnification of the framed area in (D). (F). Control of *β-catenin1* localization in male testis. (G). Low magnification showing the adult testis of a neomale. (H). High magnification of the framed area in (G). (I). Control of *β-catenin1* localization in neomale testis. Three samples were handled. Oocytes at different developmental stages are marked by I, II, III and IV. PSC: primary spermatocytes; SSC: secondary spermatocytes; LE: Leydig cells; SP: spermatid; SE: Sertoli cells. Scale bars: 100 μm.

*In situ* hybridization (ISH) showed that much higher expression of *CS-β-catenin1* occurred in the ovaries (Fig 4A and 4B) than in the testis of males and neomales (Fig 4D, 4E, 4G and 4H). In addition, ovaries contain oocytes at different developmental stages (stages I-IV). We observed the strongest hybridization signals in the stage I, II and III oocytes, and faint signals were detected in stage IV oocytes (Fig 4A and 4B). Compared to the ovary, there was relatively little expression in spermatids and sperms, and no positive signals were found in the Sertoli cells of the testis of males (Fig 4D and 4E). ISH revealed the expression of mRNA *CS-β-catenin1* in the testis of neomales; weak signals in the spermatogonium, primary spermatocytes and Sertoli cells (Fig 4G and 4H). In the negative controls, no specific signals were detected (Fig 4C, 4F and 4I).

### *CS-β-catenin1* and associated genes in ovaries treated with quercetin

**Fig 5:**
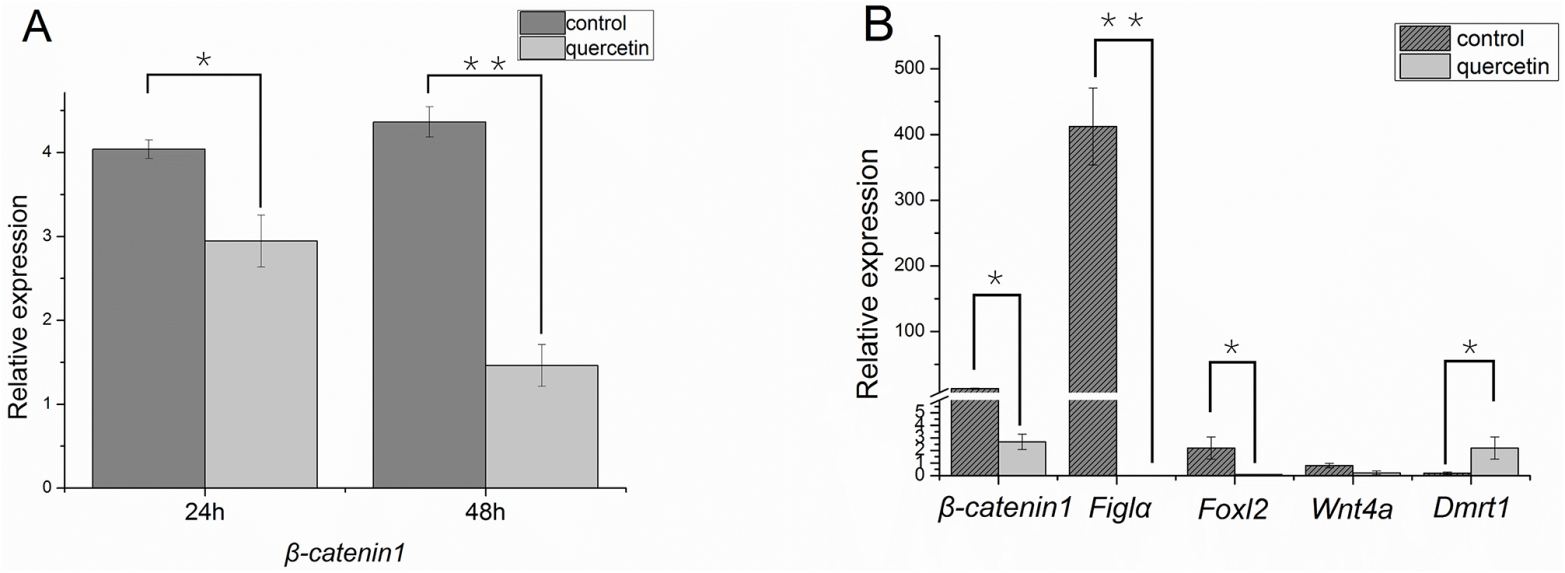
Relative mRNA expression levels of *CS-β-catenin1* and other sex-related genes after treatment with quercetin. (A) Expression of *CS-β-catenin1* 24 and 48 h after injection with quercetin. (B) Expression of *CS-β-catenin1*, *CS-Figla*, *CS-Foxl2*, *CS-Wnt4a* and *CS-dmrt1* 48 h after injection with quercetin. The expression of the *β-actin* gene was used as an internal control. Three samples were handled. Values with asterisks indicate a significant difference (*P*<0.05).

The transcription of the *CS-β-catenin1* can be significantly suppressed by quercetin, and the treatment time was also determined as 48 h by comparing the suppression effects (Fig 5A). After 48h, all fish were health and no adverse effects were experienced. After *CS-β-catenin1* suppression, the expression of *CS-Figla* rapidly reduced to almost undetectable levels, *CS-foxl2* declined in the ovaries (Fig 5B), while *CS-dmrt1* was up-regulated (Fig 5B).

### Methylation patterns in ovaries, testis and neomale gonads

**Fig 6:**
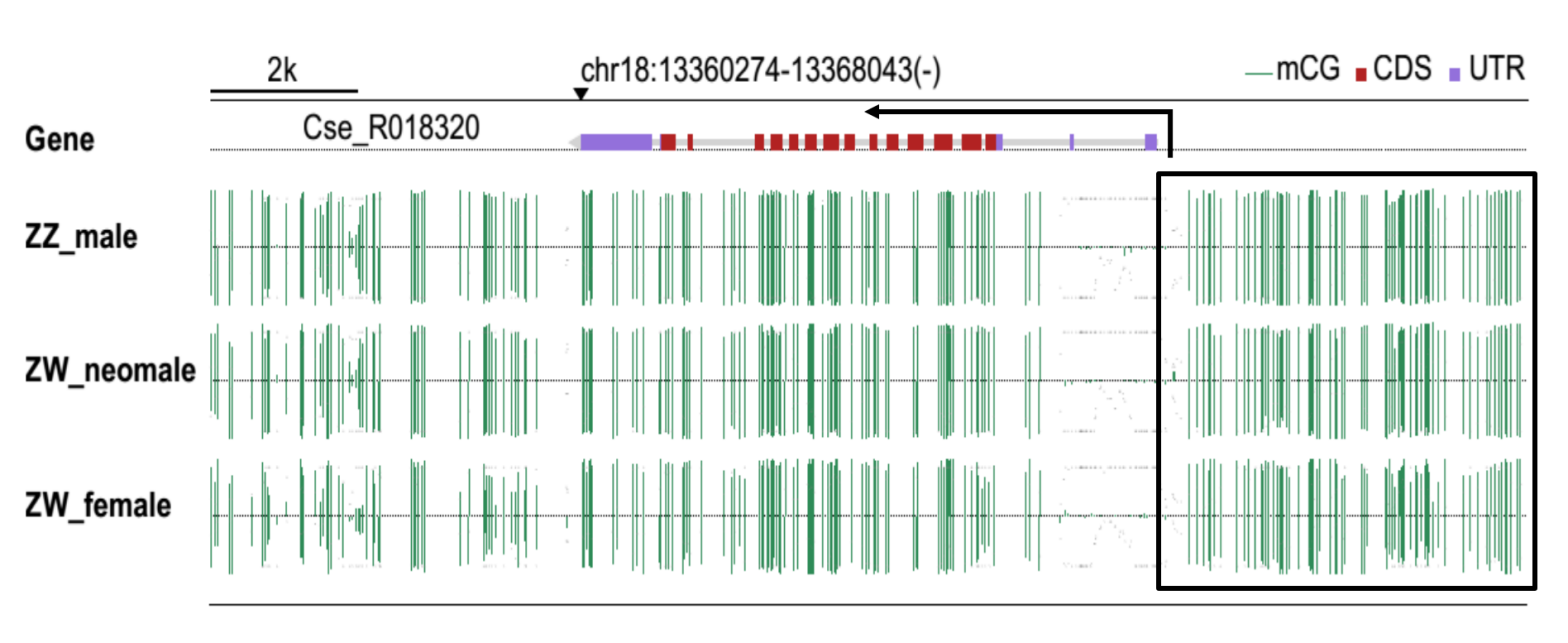
Methylation analyses of *CS-β-catenin1* in gonads. The *CS-β-catenin1* genomic sequence with gene body, also the 2kb up-and downstream area was analyzed. The methylation level of cytosines is shown with green vertical lines. The boxed area indicated the not different significantly methylation sites distributed in the upstream of 5’UTR (about 2 kb) of *CS-β-catenin1* gene. Arrows indicate direction of transcription.

Among females, males and neomales, the location of the 4 kb area upstream of ATG and the *CS-β-catenin1* gene body showed no significant methylation in the putative region between females, males and neomales (Fig 6).

## Discussion

Although the *β-catenin* gene has been reported in a few teleost species, systematic studies of its potential function are limited. In the present study, for the first time, the *β-catenin1* gene was cloned and characterized in *C*. *semilaevis*, This was named *CS-β-catenin1*, and its expression was analysed as well as the promoter methylation pattern in the gonads. By using an inhibitor of *β-catenin1* gene expression *in vivo*, we showed that *CS-β-catenin1* was a potential regulator of *CS-Figla* expression in the ovaries.

Two different *β-catenin* genes, *β-catenin1* and *β-catenin2*, were identified in the genome of teleosts, which may have arisen from whole-genome duplication [29]. Based on the evolutionary conservation of the teleost genome, we speculated that the *β-catenin2* gene probably exists in *C*. *semilaevis*, and further studies are needed to confirm our speculations.

In this present study, *CS*-β-catenin1 was shown to contain 12 ARM repeat regions, which are highly conserved among the species. Similarly, the conserved ARM regions were found in *Danio rerio*, *Nile tilapia*, *Homo sapiens* and *Chlamys farreri* [6, 11, 12, 27]. These regions are thought to be involved in the binding of β-catenin to Axin, glycogen synthase kinase 3β (GSK3β) and adenomatous polyposis coli (APC), which are three key molecules in the canonical Wnt pathway. Thus, these regions play a central regulatory role in this pathway [30–32]. In addition, these regions are thought to be required for β-catenin to interact with cell adhesion molecule E-cadherin, the tumour-suppressor gene adenomatous polyposis coli (APC) and α-catenin, as well as for the mediation of adherens junctions of the plasma membrane to the cytoskeleton [33]. We expect that the ARM regions from *CS-β-catenin1* will have similar functions.

In *C*. *semilaevis*, histological gonad differentiation was found to begin at 56-62dph, and testicular and ovarian differentiation do not occur simultaneously. Ovarian differentiation begins earlier than testicular differentiation with the appearance of an ovarian cavity. After the cavity has formed, the testes start to differentiate [20]. In our study, we observed clear *CS*-*β-catenin1* mRNA expression at 48 dph, which occurred just before the histological differentiation of the ovaries. Based on these results, we hypothesize that *CS-β-catenin1* is involved in ovary growth. Moreover, CS-*β-catenin1* probably sustained its expression until 2yph in the adult ovaries.

The cyto-locations of *β-catenin* in the gonads vary among species. In mice, *β-catenin* mRNA is mainly expressed in ovarian somatic cells, such as granulosa cells and surface epithelium cells [34]. In *C*. *semilaevis*, *Nile tilapia* and *Chlamys farreri*, *β-catenin* is predominately expressed in the oocytes, especially in the early stages of development [12, 27]. However, chicken *β-catenin* mRNA is transcribed only in the Sertoli cells of the testes from the immature to mature stages [34]. We speculate that this restricted transcription is probably caused by differences in the species. Additionally, it is likely related to the functional diversity of *β-catenin* among different cell types. In mice, *β-catenin* is expressed in the testes [7]. However, mutations affecting *β-catenin* expression did not result in phenotypic changes in the testes [7], which suggested that *β-catenin* is probably not important for testis development in mammals. In the present study, we also observed weak *β-catenin* expression in the germ cells and somatic cells of testes in 1 yph *C*. *semilaevis*, and there was no significant expression fluctuations during the development of testis. We speculate that, as in mammals, *β-catenin* is likely not necessary for testicular development in *C*. *semilaevis*.

Wnt4 is the key signalling molecule in the canonical Wnt/β-catenin pathway, which has important roles in embryonic development and disease, tissue self-renewal, and adipogenesis of vertebrates [35–38]. Additionally, the Wnt4/β-catenin pathway is associated with gonad development in mammals [39, 40] and teleosts, such as *Acanthopagrus schlegelii* and zebrafish [41, 42]. For example, the absence of Wnt4 protein results in female to male sex reversal and promotes the development of male reproductive ducts in mice with XX gonads [10]. In our previous studies, we identified a homologous gene of *Wnt4* (*Wnt4a*) and found that it was predominately expressed in oocytes in the ovaries, and in male germ cells, it was mainly expressed in the testes [43]. Furthermore, in the present study, *CS-β-catenin1* showed a similar expression pattern as *Wnt4a*. Our results, together with the reports above, indicate that the canonical Wnt4/β-catenin pathway likely exists in *C*. *semilaevis* and plays an important role in gonad development. We speculate the Wnt4/β-catenin1 pathway is likely to be conserved and is an ancient pathway in the determination of gonad development and differentiation in vertebrates.

The regulatory roles of β-catenin in gonad development have been studied extensively in vertebrates [44–46]. In teleosts, however, the regulatory function of *β-catenin* during gonad development is still poorly understood. In *C*. *semilaevis*, *Dmrt1* was identified as a testis-related candidate gene which is important in sex determination of males [20], while *Figla* was suggested to be a female-related gene that was involved in ovary differentiation, i.e., folliculogenesis [47–49]. Additionally, *foxl2* had higher expression in female gonads [50]. In addition, *Wnt4a* was regarded as a potential gene for sex reversal [43]. To investigate the regulatory pathway of *β-catenin* in *C*. *semilaevis*, we measured the expression of *dmrt1*, *foxl2*, *wnt4a* and *Figla* after *in vivo* knockdown of *β-catenin* in the ovaries. Compared to the expression of *dmrt1*, which significantly increased, the expression of *Figla* and *foxl2* displayed an opposite pattern and markedly decreased. Therefore, we speculated that the normal physiological functions of *β-catenin1* in the ovaries were likely related to promoting female-related gene expression and repressing male-related gene expression in *C*. *semilaevis*, which was similar to findings in *Nile tilapia* [12]. *Figla* was deemed to favour ovarian differentiation by antagonizing spermatogenesis in *Nile tilapia* [51]. However, differences appeared in the expression of *foxl2* after knocking out *β-catenin* in *Nile tilapia*. When *β-catenin* was knocked out, *foxl2* expression increased, which suggested that there was a possible compensatory effect for the deficiency. We hypothesized that other pathways would exist to compensate for the lack of *β-catenin1* in addition to *foxl2* in the development of ovaries in *C*. *semilaevis*.

DNA methylation, especially in the promoter region, is a heritable epigenetic modification that plays a crucial role in the modification of phenotypes and the regulation of gene expression [52, 53]. In *C*. *semilaevis*, many sex-related genes are regulated by DNA methylation, including the gametogenesis-related genes *Altesk1*, *piwil2*, *wnt4a* and *neurl3* [43, 54–56], as well as the gonad differentiation-related genes *GATA6* and *GATA4* [57, 58]. To explore the reasons why there was differential *β-catenin1* mRNA expression among the gonads in different sexes of *C*. *semilaevis*, we analysed the degree of methylation of *CS*-*β-catenin1* in males, females and neomales in the promoter region, but no significant results were found. This indicates that *β-catenin1* transcription is not primarily regulated by differential methylation between testis and ovaries.

## Conclusion

In summary, the *β-catenin1* gene was successfully isolated from *C*. *semilaevis*. *CS-β-catenin1* displayed an expression pattern that was predominantly in the gonads and the germ cells of the ovaries, which suggested that it has a potential function in ovarian germ cells. In addition, we showed that the normal physiological functions of *CS-β-catenin1* in the ovaries were to promote female-related gene expression and repress male-related gene expression. However, the molecular mechanism of *CS-β-catenin1* function is unclear, and further studies are needed.

## Supporting information

S1 Fig Protein sequence and structure of *CS-β-catenin1*. (A) Deduced protein sequence of *CS-β-catenin1*. The predicted ARM domain containing 12 repeats is marked with red and blue text. (B) Schematic illustration of *CS*-β-catenin1 structure.
(TIF)

S1 Table The accession numbers for multiple alignment and construction phylogenetic tree obtained from GenBank. (DOC)

S2 Table Primers and primer sequences used in this study. (DOC)
