# Supplementary figures and images for: Identification and analysis of the β-catenin1 gene in half-smooth tongue sole (Cynoglossus semilaevis)
Source: PLoS One. 2017 May 10;12(5):e0176122. doi: 10.1371/journal.pone.0176122 (PMC5425175; doi:10.1371/journal.pone.0176122)

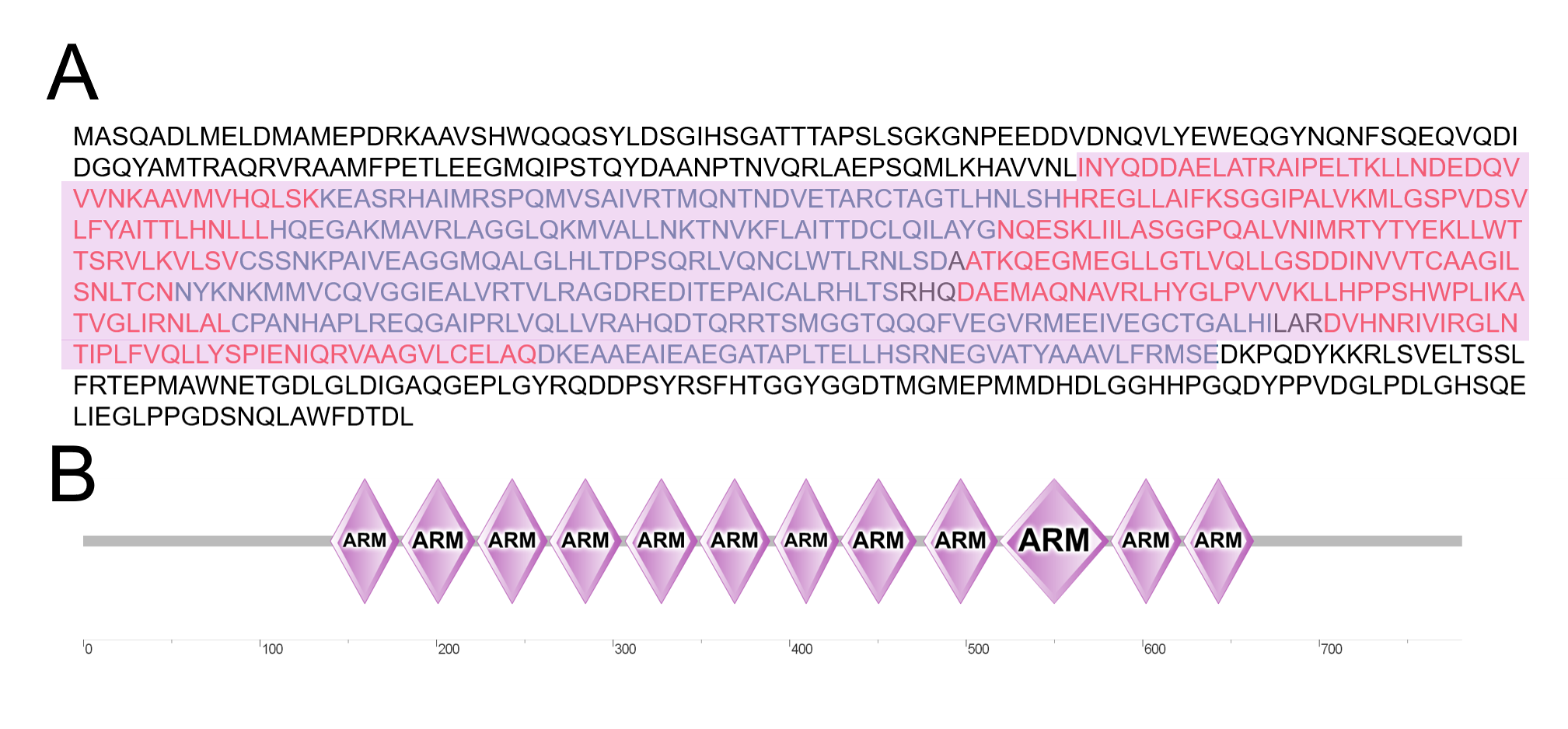

Supplement: S1 Fig — (A) Deduced protein sequence of CS-β-catenin1. The predicted ARM domain containing 12 repeats is marked with red and blue text. (B) Schematic illustration of CS-β-catenin1 structure. (TIF) [file pone.0176122.s001.tif]
