# Supplementary material for: Identification and analysis of the β-catenin1 gene in half-smooth tongue sole (Cynoglossus semilaevis)
Source: PLoS One. 2017 May 10;12(5):e0176122. doi: 10.1371/journal.pone.0176122 (PMC5425175; doi:10.1371/journal.pone.0176122)
Supplement: S1 Table — (DOC) [file pone.0176122.s002.doc]

**Table 1**

| Species name | Genbank accession number |
| --- | --- |
| *C.semilaevis* | KX898023 |
| *Larimichthys crocea* | KKF08667.1 |
| *Carassius auratus* | ACI02123.1 |
| *Danio rerio* | AAM53438.1 |
| *Salmo salar* | ACN10972.1 |
| *Pelodiscus sinensis* | BAD74125 |
| *Anas platyrhynchos* | NP_001297337.1 |
| *Gekko japonicus* | AHB33197.1 |
| *Homo sapiens* | CAA61107 |
| *Gallus gallus* | AAB80856 |
| *Mus musculus* | NP_001159374 |
| *Ovis aries* | ACU45489.1 |
| *Sus scrofa* | ACA03158.1 |
