# Supplementary material for: Identification and analysis of the β-catenin1 gene in half-smooth tongue sole (Cynoglossus semilaevis)
Source: PLoS One. 2017 May 10;12(5):e0176122. doi: 10.1371/journal.pone.0176122 (PMC5425175; doi:10.1371/journal.pone.0176122)
Supplement: S2 Table — (DOC) [file pone.0176122.s003.doc]

**Table 2**

| Primer | Sequence(5’-3’) | Usage |
| --- | --- | --- |
| *CS-β-catenin1*- F | AGGCAACCCTGAGGAGGACGA | 5’ or 3’RACE |
| *CS-β-catenin1*- R | ACTGGAGCAGACTGACAGCACTTT | 5’ or 3’RACE |
| *CS-β-catenin1*-5’GSP | CTGGTTGTAGCCCTGCTCCCACTCAT | 5’region clone |
| *CS-β-catenin1*-5’NGSP | CATCGTCCTCCTCAGGGTTG | 5’region clone |
| *CS-β-catenin1*-3’GSP | GGGCATATGGCAACCAGGAAAGCAAAT | 3’region clone |
| *CS-β-catenin1*-3’NGSP | GGACCACAAGCAGAGTTCTCAAA | 3’region clone |
| *CS-β-catenin1*-RT-F | CAGGAGGGAATGGAAGGTTTG | qRT-PCR |
| *CS-β-catenin1*-RT-R | CTAATGCCTCAATACCGCCAAC | qRT-PCR |
| *CS-β-catenin1*-ISH-F | TCAGAATTCTATTCTATGCCATCACC | ISH |
| *CS-β-catenin1*-ISH-R | TCCAAGCTTAGTAGTCCTGAGTTGATA | ISH |
| *CS-Dmrt1-*F | CCGGACGGCTTCGTGTC | qRT-PCR |
| *CS-Dmrt1-*R | CTTCCACAGGGAGCAGGCAGT | qRT-PCR |
| *CS-Figla*-F | AAGACGGTTTGTATTTCGTAGC | qRT-PCR |
| *CS-Figla*-R | CTAGACCTTCTGTAGATGTGGC | qRT-PCR |
| *β-actin*-F | CCTTGGTATGGAGTCCTGTGGC | qRT-PCR |
| *β-actin*-R | TCCTTCTGCATCCTGTCGGC | qRT-PCR |
